# Apelin and energy metabolism

**DOI:** 10.3389/fphys.2015.00115

**Published:** 2015-04-10

**Authors:** Chantal Bertrand, Philippe Valet, Isabelle Castan-Laurell

**Affiliations:** ^1^Institut National de la Santé et de la Recherche Médicale, U1048Toulouse, France; ^2^Institut des Maladies Métaboliques et Cardiovasculaires (I2MC), Université Toulouse III—Paul SabatierToulouse, France

**Keywords:** obesity, type 2 diabetes, apelin, adipokine, insulin sensitivity

## Abstract

A wide range of adipokines identified over the past years has allowed considering the white adipose tissue as a secretory organ closely integrated into overall physiological and metabolic control. Apelin, a ubiquitously expressed peptide was known to exert different physiological effects mainly on the cardiovascular system and the regulation of fluid homeostasis prior to its characterization as an adipokine. This has broadened its range of action and apelin now appears clearly as a new player in energy metabolism in addition to leptin and adiponectin. Apelin has been shown to act on glucose and lipid metabolism but also to modulate insulin secretion. Moreover, different studies in both animals and humans have shown that plasma apelin concentrations are usually increased during obesity and type 2 diabetes. This mini-review will focus on the various systemic apelin effects on energy metabolism by addressing its mechanisms of action. The advances concerning the role of apelin in metabolic diseases in relation with the recent reports on apelin concentrations in obese and/or diabetic subjects will also be discussed.

## Introduction

Nutrient metabolism and energy homeostasis are tightly regulated by endocrine, paracrine, and autocrine factors. Moreover, skeletal muscle, liver, adipose tissue and pancreatic β-cells play a major role in the maintenance of energy balance. A rapid modification of energy balance leads to obesity that in turn is a crucial cause of insulin resistance. Several mechanisms linking obesity to insulin resistance have been proposed. Among them, adipocyte-secreted factors or adipokines have been shown to play an important role. Alteration of their production (excess or deficit) could directly promote or delay the onset of insulin resistance. The role of leptin and adiponectin has been extensively studied (for review see Lafontan and Viguerie, 2006; Tishinsky et al., 2012; Farooqi and O'Rahilly, 2014). In 1998 Tatemoto et al identified apelin as the ligand of the APJ receptor, a G protein coupled receptor (Tatemoto et al., 1998). Apelin gene encodes for a 77 amino acid preproprotein and the apelin propeptide contains several doublets of basic amino acids implicating potential proteolytic cleavage sites for endopeptidases which give rise to several bioactive carboxy-terminal fragments including apelin-36, apelin-17, and apelin-13 but also the pyroglutamate apelin-13 which is protected from exopeptidase degradation (Masri et al., 2005). The group of Tatemoto has described the presence of apelin in rat adipose tissue (Wei et al., 2005) but the actual secretion from human and murine adipocytes was reported by Boucher et al. demonstrating that apelin was a new adipokine (Boucher et al., 2005). Apelin and its receptor APJ are widely expressed in several tissues (stomach, heart, lung, skeletal muscle, etc.) and in different regions of the brain, including the hypothalamus (O'Carroll et al., 2013). The apelin/APJ system is involved in a wide range of functions. Neither the effects of apelin on the regulation of cardiac and vascular functions, fluid homeostasis and angiogenesis, (Chapman et al., 2014) nor its central actions on energy metabolism (Knauf et al., 2013) are presented in this article since these aspects were recently reviewed. This mini-review will discuss the recent advances concerning the role of apelin on energy metabolism particularly in pathophysiological situations (obesity, type 2 diabetes) and will try to establish a link between plasma apelin concentrations and metabolic diseases in humans.

## Apelin and glucose metabolism

One of the first apelin effects observed on glucose metabolism, apart from that on insulin secretion (Sorhede Winzell et al., 2005), is its glucose-lowering effect both in fasted conditions and during a glucose tolerance test (Dray et al., 2008) in standard mice. This decreased glycemia has been shown to be mainly due to increased glucose uptake in target tissues such as skeletal muscle and adipose tissue (Dray et al., 2008). Since the muscles represent the main entry of glucose, apelin effect was studied in isolated soleus muscle. Apelin stimulated glucose transport and its effect was additive to that of insulin (Dray et al., 2008). The signaling pathway was depicted and it was shown that apelin stimulated the phosphorylation of the AMP-activated protein kinase (AMPK) but also the endothelial NO synthase (eNOS) (Dray et al., 2008). The importance of both enzymes has been demonstrated by the use of eNOS^−/−^ mice and DN-AMPK mice (muscle-specific inactive AMPK) respectively. Later on, the study of Yue et al. also reported that apelin was able to stimulate glucose transport *in vitro* in C2C12 muscle cells through a pathway involving AMPK but not eNOS (Yue et al., 2010). This discrepancy could be due to the fact that NOS inhibitors were used in the study of Yue et al. and that these inhibitors are efficient to decrease glucose uptake *in vivo* in muscle cells but not *in vitro* as previously reported (Roy et al., 1998). In addition, apelin also increased Akt phosphorylation in muscle manner both *ex vivo* (Dray et al., 2008) and *in vitro* (Yue et al., 2010). Interestingly, apelin is still able to stimulate glucose uptake in muscle of obese and insulin-resistant mice. This leads to an overall better insulin sensitivity (Dray et al., 2008). The role of apelin in glucose homeostasis was confirmed by the phenotype of apelin null (apelin^−/−^) mice exhibiting hyperinsulinemia and insulin resistance (Yue et al., 2010). The loss of insulin sensitivity in apelin^−/−^ mice was exacerbated by a high fat/ high sucrose diet (Yue et al., 2010).

Even though apelin-induced glucose transport has not yet been reported in isolated mouse adipocytes, apelin stimulates glucose transport in an AMPK-dependent way in human adipose tissue explants (Attane et al., 2011). This has also been observed in 3T3-L1 adipocytes through a mechanism dependent on PI3K/Akt activation but the role of AMPK was not studied (Zhu et al., 2011). In addition, in insulin-resistant 3T3-L1 cells (due to TNFα treatment), apelin increases the insulin-stimulated glucose transport (Zhu et al., 2011). Skeletal muscle and adipose tissue are not the only tissues where apelin stimulates the entry of glucose. *In vivo*, apelin has been shown to increase myocardial glucose uptake and Glut4 membrane translocation in C57BL/6J mice (Xu et al., 2012). Apelin also increases glucose transport *in vitro*, in H9C2 cardiomyoblasts (Xu et al., 2012). A role of apelin has also been shown in intestinal glucose absorption. Ingested glucose can rapidly induce the secretion of apelin in the intestinal lumen in mice (Dray et al., 2013). This study also shows that, when apelin is administered orally, the amount of glucose transporters SGLT1 is decreased in enterocytes, whereas that of Glut2 is increased due to AMPK activation. This results in an increased intestinal absorption of glucose. These data suggest that glucose arrival in the intestine causes its own absorption by inducing the paracrine secretion of apelin. A transient increase in blood glucose levels in the portal vein could induce rapid secretion of insulin (Fukaya et al., 2007), and an improved insulin sensitivity (Burcelin et al., 2000; Delaere et al., 2010). Thus, apelin could also regulate glucose metabolism, by promoting glucose absorption by the enterocytes and then by increasing portal blood glucose and insulin secretion. This could be in agreement with the fact that apelin was shown to increase GLP-1 secretion (Wattez et al., 2013).

Although all studies did not report a significant decrease in fasting blood glucose in obese and insulin resistant mice in response to apelin, decreased insulinemia has frequently been observed. This may be the result of improved insulin sensitivity or a direct effect of exogenous apelin on the pancreas. Accordingly, apelin was shown to decrease insulin secretion stimulated by different glucose concentrations (Guo et al., 2009; Ringstrom et al., 2010). Thus, by activating AMPK and bypassing insulin signaling, apelin exerts direct anti-diabetic effects, which could have an important impact in insulin resistant conditions.

## Apelin and lipid metabolism

Few publications describe acute effects of apelin on lipid metabolism. In both isolated adipocytes and differentiated 3T3-L1 adipocytes, apelin was shown to inhibit isoproterenol (β-adrenergic agonist) -induced lipolysis through a pathway involving Gq, Gi, and AMPK (Yue et al., 2011). These results were confirmed by Than et al. (2012), who showed that apelin decreases the release of FFA by 3T3-L1 adipocytes through AMPK activation and by increasing the amount of perilipin surrounding the lipid vacuoles, giving them a greater stability and a resistance to lipases (Than et al., 2012). However, in human adipose tissue explants or human isolated adipocytes, apelin had no effect on basal or isoproterenol-stimulated lipolysis (Attane et al., 2011). Effects on adipose tissue and lipolysis were also found *in vivo* after a chronic apelin treatment in standard or high-fat diet (HFD) fed mice. Indeed, Higuchi et al. showed that daily ip apelin injections during 2 weeks decrease the triglycerides content in adipose tissue and the weight of different fat depots in standard mice and in HFD fed mice (Higuchi et al., 2007). Similar results were obtained in transgenic mice over-expressing apelin (Tg-apelin mice) fed a HFD (Yamamoto et al., 2011).

Chronic apelin treatment, in obese and insulin resistant mice, was also shown to increase fatty acid oxidation in muscles through AMPK activation (Attane et al., 2012). More recently, chronic apelin treatment has also been shown to prevent reduction of fatty acid and glucose oxidation in a model of obesity-related decline of cardiac function (Alfarano et al., 2015). In addition to stimulate the utilization of lipids, apelin treatment increases mitochondrial biogenesis in skeletal muscle (Attane et al., 2012) and cardiomyocytes (Alfarano et al., 2015) by a mechanism involving increased expression of peroxisome proliferator-activated receptor γ co-activator 1α (PGC1α). Increased mitochondrial DNA content in skeletal muscle was also found in Tg-apelin mice (Yamamoto et al., 2011) in agreement with the effect of apelin on mitochondrial biogenesis. Interestingly, the resistance to obesity of Tg-apelin mice was correlated with an increase in vessel formation in skeletal muscle. The importance of vessels integrity in both blood and lymph vasculature was recently confirmed in apelin^−/−^ mice (Sawane et al., 2013). Indeed, weight gain observed in apelin^−/−^ mice could be related to increased vascular permeability that in turn would induce greater fatty acids uptake in adipose tissue (Sawane et al., 2013). Thus, apelin might also prevent development of obesity through the maintenance of vascular integrity.

Energy expenditure in response to apelin treatment has also been studied via thermogenesis. Higuchi et al. reported that rectal temperature and O_2_ consumption were higher in apelin-treated chow-fed mice probably mediated by the increased expression of mitochondrial uncoupling protein 1 (UCP1) observed in brown adipose tissue (Higuchi et al., 2007). O_2_ consumption and body temperature were also increased in HFD fed Tg-apelin mice (Yamamoto et al., 2011) but not in obese and insulin resistant mice in response to chronic apelin treatment (Attane et al., 2012). However, food intake was not altered in both models.

All together, these studies clearly show that apelin, by itself, exerts metabolic functions such as glucose uptake but also improves insulin sensitivity since, for example, at the end of chronic apelin treatment, insulin-induced glucose transport was increased in skeletal muscles and there is an overall better insulin and glucose tolerance (Attane et al., 2012). Therefore apelin could be proposed as an interesting therapeutic target in the treatment of type 2 diabetes.

## Changes in apelin concentrations related to human metabolic diseases

Numerous studies have reported increased plasma apelin concentrations in obese and/or diabetic subjects (for review see Castan-Laurell et al., 2011). Apelin-17 and [pyr-1]-apelin-13 may represent the predominant forms in plasma (De Mota et al., 2004; Azizi et al., 2008). During the last years, additional information was provided by assays performed especially in diabetic patients and in patients involved in weight loss intervention studies. Interestingly, plasma apelin has been shown to be a novel biomarker for predicting diabetes in Han Chinese subjects (Ma et al., 2014). Plasma apelin concentrations were higher in women than in men but they were associated with a risk of diabetes only in men (Ma et al., 2014). Recent data have also shown that apelin concentrations were significantly higher in type 1 diabetic patients than in control subjects and even higher than in type 2 diabetic patients (Habchi et al., 2014), in line with the previous study of Alexiadou et al. focused on type 1 diabetic subjects (Alexiadou et al., 2012). All together these studies pointed out the role of systemic apelin in metabolic diseases. What is the meaning of elevated apelinemia? Is obesity a main determinant of elevated plasma apelin concentration? Different elements could be provided. Habchi et al. demonstrated that serum apelin levels were negatively correlated with glycosylated hemoglobin in type 2 diabetic patients, suggesting that circulating apelin is associated with better glycaemic control (Habchi et al., 2014). Increased concentrations of apelin in type 1 diabetes could be an attempt to compensate for the lack of insulin and to overcome insulin resistance. However patients were also treated with insulin, which could as well explain this rise, since insulin is one of the most important regulator of apelin expression and secretion (Boucher et al., 2005). Moreover, type 1 diabetic patients are not obese, suggesting that obesity is probably not the main determinant of increased apelin levels. In line with this point, an absence of correlation between plasma apelin concentrations and BMI has often been described (Castan-Laurell et al., 2011). The recent study of Krist et al. also gives further insights. They aimed to investigate whether changes in circulating apelin, in a context of weight loss, are primarily due to a reduced body fat mass or reflect the improved insulin sensitivity. First, all the different weight loss intervention studies (hypocaloric diet, bariatric surgery or exercise program) reduced the elevated serum apelin concentration determined in different cohorts of obese and diabetic patients as previously reported (Heinonen et al., 2009; Castan-Laurell et al., 2011). Secondly, significant BMI-independent correlations between reduced apelin levels and improved insulin sensitivity were found (Krist et al., 2013). Thus, it could be hypothesized that the increased plasma apelin observed in type 2 diabetic patients, is, as in type 1 diabetes, a compensatory mechanism devoted to directly decrease insulin resistance since apelin exerts different metabolic actions itself. When insulin resistance is decreased, this may lead to decreased apelin levels. It has thus been proposed that lower apelin serum concentrations in healthy lean individuals may be a consequence rather than a cause of normal insulin sensitivity (Krist et al., 2013).

## Conclusion

**Figure 1:**
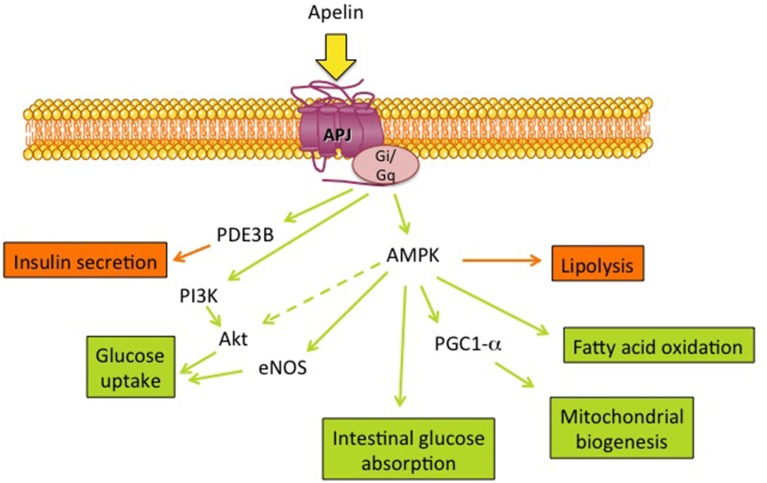
**Metabolic effects of apelin and its main signaling targets**. Apelin, the ligand of the G protein coupled receptor APJ, can stimulate several metabolic functions (green arrows/boxes) and inhibit (orange arrows/boxes) lipolysis as well as insulin secretion through different signaling pathways: PDE3B, phosphodiesterase 3B; AMPK, AMP-activated protein kinase; PGC1-α, peroxisome proliferator-activated receptor γ co-activator 1α; eNOS, endothelial NO synthase; PI3K, phosphatidylinositol 3-kinase and Akt.

The metabolic effects of apelin (Figure 1) described in different mouse models (diet-induced obesity, transgenic models) have underlined the beneficial roles of apelin on both energy metabolism and insulin sensitivity. Still, there remain many questions and many tools need to be developed. Long term apelin treatment studies, in both healthy and pathological conditions, need a more integrative view including cardiac, vascular and central effects. The methods used for apelin quantification include enzyme immunoassays and radioimmunoassays but give a wide range of basal values depending on the studies. More reliable assays, easy to use, are necessary. It will also be important to know whether the elevated serum apelin concentrations correspond to active apelin and what are the predominant forms of apelin in metabolic diseases and their variations. Finally, selective agonists and antagonists for APJ started to be developed but they need to be tested on metabolic tissues and their signaling more largely described. All these points are important in order to validate the promising anti-diabetic properties of apelin.

### Conflict of interest statement

The authors declare that the research was conducted in the absence of any commercial or financial relationships that could be construed as a potential conflict of interest.
